# Evaluating the Effectiveness of a Creative Arts and Philosophical Inquiry Intervention Rooted in Self-Determination Theory to Promote Adaptive Coping with Eco-Anxiety among Elementary School Children: A Pilot Randomized Cluster Trial

**DOI:** 10.1177/24705470261442334

**Published:** 2026-04-17

**Authors:** Terra Léger-Goodes, Catherine M. Herba, Jonathan Smith, David Lefrançois, Marc-André Éthier, Jasmine Piché, Catherine Malboeuf-Hurtubise

**Affiliations:** 1 Department of Psychology, 14845 Université du Québec à Montréal (UQAM), Montreal, Canada; 2 Azrieli Research Center of the CHU Sainte-Justine, Montreal, Canada; 3 Department of Preschool and Primary Education, 7321 Université de Sherbrooke, Sherbrooke, Canada; 4 Centre de recherche du Centre Hospitalier de l’Université de Sherbrooke, Sherbrooke, Canada; 5 Department of Educational Sciences, Université du Québec enOutaouais, Saint-Jérôme, Canada; 6 Department of Didactics, 5622 Université de Montréal, Montréal, Canada; 7 Department of Psychology, 7321 Université de Sherbrooke, Sherbrooke, Canada; 8 School of Psychology, Université Laval, Quebec City, Canada; 9 Canada Research Chair on Arts-Based and Existential Interventions in Youth Mental Health, Québec, Canada

**Keywords:** climate change, eco-anxiety, coping with climate change, art-based intervention, philosophical inquiry, self-determination, existential psychology, positive psychology

## Abstract

**Background:**

Children's exposure to climate change information through educational and media channels can lead to experiencing eco-anxiety. When children use maladaptive coping strategies such as avoidance and de-emphasizing the seriousness of the threat, their anxiety and distress levels increase significantly. Research indicates that creative arts and existential psychology interventions grounded in self-determination theory may promote healthier coping mechanisms like creating meaning.

**Methods:**

This randomized cluster pilot study examined whether a creative arts and philosophical inquiry intervention could foster adaptive coping strategies in elementary school children. Eighty-seven students across four classrooms participated. The experimental group (*n* = 46) received a seven-week intervention addressing eco-anxiety themes, while control groups (*n* = 41) remained on a waiting list. Pre- and post-intervention questionnaires assessed climate change coping strategies, eco-anxiety dimensions, and self-determination.

**Results:**

Results showed significant decreases in overall eco-anxiety, affective eco-anxiety, and rumination eco-anxiety scores from pre- to post-intervention, regardless of group assignment. No significant interaction effects emerged between the intervention and time on any of the measured variables, indicating the intervention did not produce differential outcomes compared to the control condition.

**Implications:** Results suggest that discussing climate change in the classroom through artistic creation and philosophical inquiry did not promote adaptive coping nor impact eco-anxiety. The observed reductions in eco-anxiety across both groups could reflect external factors or contamination effects rather than intervention effectiveness. Future research should employ longitudinal designs with larger, more diverse samples and incorporate more sensitive measurement tools, shorter questionnaires, and child-appropriate assessments to better understand intervention impacts on children's climate-related mental health.

## Introduction

### Background

#### Eco-Anxiety in Children

Children are increasingly aware of the impacts of climate change. Not only are they more exposed to information about their manifestations and consequences, but they may also witness them firsthand, providing fertile ground for developing eco-anxiety.^1,2^ Many children experience a wide range of emotions that constitute eco-anxiety, including fear for their future, sadness for the loss of biodiversity, and anger towards previous generations.^3,4^ Boivin et al (2025) proposed a comprehensive definition of eco-anxiety:

A sociopsychological state characterized by fluctuating feelings of worry, distress, and apprehension in response to the observed and anticipated effects of climate change and environmental disruptions, as well as their impacts on people's way of life and the perceived inadequacy of societal responses and policies. (p. 9)^5^

Although eco-anxiety is considered a normal response to a truly upsetting situation,^6^ some people may experience high levels of distress.^7^ Indeed, in certain cases, people can experience a range of symptoms, including significant worry, difficulty concentrating and sleeping.^8^ A survey of young adults aged 18 to 24 years old revealed that 49% of them experience this type of distress.^9^ Similarly, 45% of youth report that their eco-anxiety negatively affects their daily functioning.^10^ Moderate eco-anxiety may be associated with high pro-environmental behaviors, but higher levels can instead be paralyzing and not translate into action.^11^ While the rates of eco-anxiety in younger children are unclear, preventing higher levels of distress associated with learning about climate change could allow them to remain engaged with the issue through adaptive coping.

#### Coping with Eco-Anxiety

The ways in which children cope with eco-anxiety can be more or less adaptative. For example, some children cope with eco-anxiety using problem-focused approaches by getting involved in pro-environmental actions. These may provide a sense of acting according to one's values. Yet, when this is the only coping strategy used, it can place a burden of responsibility on children and has been linked to symptoms of depression and anxiety.^12^ Most children cope with eco-anxiety through avoidance and de-emphasizing the threat.^13^ However, this type of coping is associated with symptoms of anxiety and cognitive dissonance.^14^ In the long term, being able to navigate difficult emotions, feeling emotionally validated, and creating meaning within the context of climate change have been identified as more adaptive forms of coping.^12,13,15^ Indeed, meaning-focused coping, which includes reframing the problem, holding space for negative and positive emotions and creating meaning in a situation where the problem cannot be solved – as is the case with climate change, has been found to be more adaptive.^12,13^ However, there are very few empirically validated interventions to promote adaptive coping with climate change in children and prevent distress.

#### Self-Determination Theory to Guide Adaptive Coping

Self-determination theory can inform on potential ways to foster meaning-focused coping and promote the satisfaction of the three fundamental psychological needs for competence, autonomy, and affiliation in the context of climate change.^15–17^ First, the need for competence implies having an impact on one's environment and mastering certain activities. Second, the need for autonomy refers to the sense of control over one's own actions and the freedom to make choices that align with personal interests and values. Third, the need for affiliation involves the need to feel connected with others, to love, and be loved. Supporting children's satisfaction of these needs can improve their school motivation, well-being, and mental health.^18^ In the context of climate change, satisfaction of the basic psychological needs can foster pro-environmental behaviors and adaptive coping.^19^ Thus, interventions rooted in self-determination theory, which integrate the support for all three basic psychological needs, could foster adaptive coping in children and prevent high levels of eco-anxiety; however, this has not yet been evaluated to our knowledge.

#### Interventions to Promote Adaptive Coping with eco-Anxiety

Initial research with adults suggests that interventions for coping with eco-anxiety should promote emotional expression, social connection, and meaning-making using group-based approaches.^20,21^ An integrative literature review of eco-anxiety in youth also highlights the importance of promoting hope, using emotional validation, questioning binary thinking patterns, and creating meaning with this population.^22^ As such, interventions that are rooted in artistic creation and existential psychology could address these aspects.

##### Art-Based Interventions

Interventions that integrate artistic creation like painting, sculpting, photography or collage are often perceived as fun and accessible while promoting emotional regulation.^23^ In the context of climate change, photography has been investigated as a potential artistic medium to explore eco-anxiety. One study on climate change with elementary school children found that photography allowed them to express emotions when sharing their pictures with the class.^24^ Photography is indeed a form of artistic expression that can be particularly beneficial in creating meaning, promoting introspection, communication, and well-being.^25,26^ Photography can give children a voice and a lens for mutual understanding, while making spaces for the creation of collective meaning regarding a specific issue.^27^ In this sense, this approach could allow children to share their knowledge, questions, and emotions through a creative means of communication, fostering meaning-making.

Other artistic mediums can also promote adaptive coping with children. A pilot study with youth aged 16–18 years old used artistic creation (creative writing, drawing, etc.) to allow space for discussion and emotional expression around the theme of climate change.^28^ This led to an increase in motivation to discuss this topic and helped youth articulate their feelings of hope for the future.^28^ Art-based approaches create space for introspection and emotional expression through an alternate, non-verbal, means of communication.^29^ Artistic creation has been shown to be particularly beneficial in exploring sensitive topics,^30^ fostering mental health and increasing children's self-expression, feelings of empowerment, hope and optimism for the future.^23^ Using drawing, painting, sculpture or collage, for example, allows children to tell their stories, reframe their experiences, and create a sense of coherence within their lives.^31^ However, there are few studies on the use of artistic creation in the context of children's eco-anxiety and coping with climate change.

##### Philosophical Inquiry Interventions

The quest for meaning is rooted in existential psychology, which seeks to address questions pertaining to meaning in life, among others. Evidence suggests that children ponder about the meaning of life, especially when confronted with climate change.^32,33^ Philosophical inquiry within the classroom can help children co-create meaning around an existential issue and has been found to be a good proxy for existential psychology with youth. This approach creates space for group discussions on a particular subject while allowing for introspection, connection with one's values, expression of one's point of view, and creative reasoning.^34^ The aim is to expose children to challenging questions that may not have a definitive answer, and encourage them to think *by* and *for* themselves in a supportive environment.^35^ More specifically, philosophical inquiry with children takes the form of group workshops and is designed to prompt them to reflect on various themes following the presentation of a primer, such as a picture, a video, or an existential question.^35,36^ Participating in such workshops could promote the development of children's social and cognitive competencies,^37^ as well as increase feelings of autonomy and decrease anxiety symptoms in elementary school children.^38^ In the context of climate change, philosophical inquiry has been used with children to learn about climate change and has led to the creation of hope narratives.^39^ However, approaches using philosophical inquiry with children in similar contexts have been criticized for their overly cognitive and abstract nature, which may not allow for a connection to emotional experiences.^36,40^ Furthermore, there are no studies that specifically document the impacts of such interventions on children's coping and eco-anxiety.

Combining philosophical inquiry and artistic creation as complementary approaches in the classroom could allow children to explore and express their own emotional experiences with climate change and foster meaning-focused coping. While the existential and cognitive aspects of climate change are essential for allowing children to explore their beliefs, values, and points of view,^39^ combining them with artistic creation also creates space to visually represent thoughts and emotions related to climate change.^41^ By combining both approaches, the basic psychological needs for competence, autonomy and affiliation are supported, as children are encouraged to think by and for themselves, using artistic mediums with which they are comfortable, in a supportive group context. The extensive rationale for this combined intervention can be found in a previously published article by the research team.^16^

##### Universal Prevention and Promotion Programs in Schools

Schools are spaces where children are exposed to learning about climate change, particularly through the science curriculum. In fact, many teachers may feel uncomfortable teaching about climate change or fear causing anxiety in children by doing so.^42^ As such, classrooms appear to be ideal spaces to both empower children within a group of others they know, as they learn about climate change. This could also empower teachers to integrate emotional expression as they teach about climate change, promoting children's adaptive coping. A universal prevention and promotion program could benefit all children by providing them with tools to navigate learning about climate change, as most will be familiar with the issue, or eventually be exposed to, as the consequences may become more frequent. A retrospective study with young adults suggests that schools should reframe climate change education, making it more accessible and relevant to their daily lives, while also addressing students’ emotional responses to learning about it.^43^ Hence, the classroom context may reach a significant number of students as they learn about climate change, preventing high levels of eco-anxiety and fostering adaptive coping, while ensuring teachers remain engaged in environmental education.^44^ However, to our knowledge, there exists no empirically validated program published and accessible to teachers.

### Study Aims and Hypotheses

This pilot study aimed to evaluate the impact of an elementary school-based intervention combining artistic creation and philosophical inquiry on children's coping with climate change, eco-anxiety, and self-determination. It was hypothesized that the children in the experimental group would show an increase in meaning-focused coping (primary outcome), and a reduction of avoidance coping (primary outcome) compared to those in the control group. The problem-focused coping outcome was exploratory, since the intervention did not aim to increase action. The association between pro-environmental action, eco-anxiety and knowledge was not documented in elementary-school children, and many influencing factors for this specific variable have been previously identified.^45^ It was also hypothesized that children participating in the intervention would show a reduction in self-reported eco-anxiety (primary outcome, including four domains of eco-anxiety: ruminative, affective, behavioral, and impact), as well as an increase in self-determination (secondary outcome) when compared to the children in the control group. The report follows the CONSORT 2010 checklist extension for reporting a cluster randomized trial.^46^ The research project was registered at clinicaltrials.gov (ID NCT07095387).

## Methods

### Trial Design

**Figure 1.**
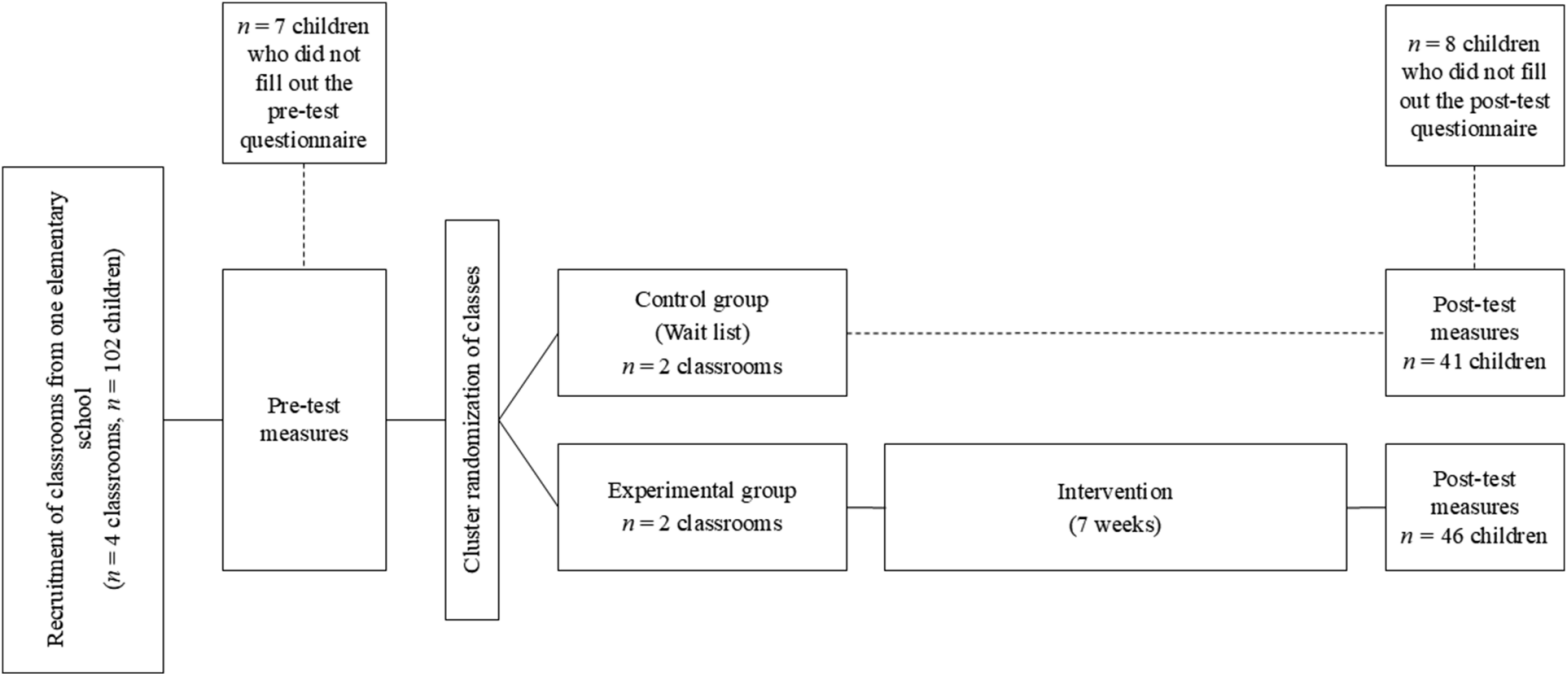
Flowchart of participants.

The present study used a pilot parallel randomized experimental cluster design, randomizing classrooms at the cluster level rather than individual participants, and comparing the experimental group to a waitlist control group. This study was conducted in a school context, in which classrooms formed pre-existing clusters that could not be divided. Classes were randomly assigned (ratio 1:1) to the experimental group (2 groups, 46 students) or the control group (2 groups, 41 students) using a random number table. Groups were considered reasonably balanced. No changes were made to the trial design after trial commencement. The flowchart of participants can be found in Figure 1.

### Participants

Sample sizes for pilot studies usually require 30% fewer participants after calculating a target sample size for continuous outcomes.^47^ An *a priori* calculation was conducted using GPower (version 3.1.9.7) for Mixed ANOVAs (repeated measures, within-between interactions) given a medium effect size (*f* = 0.25), an alpha level of 0.05, 80% power, two groups and two measurements. Since no prior study existed to determine the effect size, the benchmark suggested by Cohen (1988) was used.^48^ The calculation yielded a required sample size of 128 students. For a pilot study, a reduction of 30% indicates that as few as 89 participants would be acceptable. Other authors argue that a pilot study with at least 25 participants per treatment arm is recommended to detect medium effect sizes.^49^ The invitation to participate in the intervention was sent to three elementary schools that had previously collaborated with the research team. One school responded favorably, and four classrooms from this one school (*n* = 102 children) accepted to participate. Teachers in the control group were specifically instructed to teach as usual and not implement any of the activities outlined in the intervention. Given the nature of the intervention, there was no blinding of participants.

The elementary school was situated in the province of Quebec, Canada. Access to nature was particularly high in the affluent neighborhood where the school was located. There was a small forest next to the school, many trees in the street, and parks within walking distance. Nonetheless, it was observed that many children drove to school and did not necessarily live in the same neighborhood as the school.

The experimental group received the seven-week intervention consisting of weekly 50-min activities that combined artistic creation (30 min) and a group-based philosophical discussion (20 min) from February to April 2024. Of the children, 41 identified as girls, and 46 identified as boys, none as non-binary or gender neutral. The ages ranged from 9 to 12 years, with a mean age of 9.9 years (SD = 0.89). The participant characteristics can be found in Table 1.

**Table 1.** Descriptive Statistics of Groups.

Class	Grade Level	Group	*n* Children in Class	*n* Girls	Mean Age in Years (SD)	Total
**1**	4	Control	22	12	9.50 (0.51)	41
**2**	4	Control	19	11	9.32 (0.48)	
**3**	4	Experimental	21	11	9.43 (0.51)	46
**4**	6	Experimental	25	11	11.08 (0.40)	
**Total sample**				45	9.90 (0.89)	87

### Ethics Approval and Consent to Participate

The present study was approved by Bishop's University Research Ethics Board on January first, 2023 (File no. 102683). Informed consent was first obtained from the teachers, who agreed that the intervention was conducted in their classroom. Informed consent was obtained through a consent form sent to parents, who signed for their children. The informed assent of the children was obtained verbally during the first workshop and before questionnaire completion.

### Intervention and Procedure

The intervention consisted of a seven-week creative arts and philosophical inquiry intervention developed by the researchers.^16^ A qualitative pilot study with one class was conducted, revealing high acceptability of the intervention and positive perceived effects from both the students’ and teacher's perspectives.^50^ Each week consisted of a one-hour period devoted to an artistic creation followed by a group discussion on a philosophical theme. The breakdown of the intervention is presented in Table 2. A doctoral student in psychology (TLG), and one research assistant (JP), a bachelor student in psychology, led the intervention. They were both supervised by the principal investigator (CMH), a clinical child psychologist. All artistic materials were provided by the research team, and the teachers were given the option to participate or not during the intervention. Most of them remained in the class and participated in the workshops. The wait-list control group maintained their classes as usual; teachers were told not to conduct any of the activities their colleagues might be doing, and to teach the usual content. None of them taught about climate change during the intervention period, as it was not covered in the usual course material at the time.

**Table 2.** Breakdown of the Weekly Activities.

Week	Artistic Creation Activity	Philosophical Inquiry Theme
1	Emotion wheel drawing	Climate change
2	Drawing of the planet in 50 years	Hope and despair
3	Photovoice: The beauty of nature	Beauty
4	Sculpture of something impressive in nature	The strength of nature
5	Photovoice: Climate change in my environment	Responsibility
6	Drawing on a rock: something I would like to take care of in nature	Taking care
7	Poster: my climate change slogan (drawing, collage, painting)	Change

One week before the intervention began, all students completed a set of questionnaires during class time on a single day. Twenty to 40 min were necessary for completion. A research assistant read the questions aloud and children could ask for clarification if needed. The same procedure was repeated one week after the intervention ended, before the wait-list control group began receiving the intervention.

### Outcomes

The following scales were used to measure coping with climate change, eco-anxiety, and self-determination. Graphic depictions of the Likert scales were added to help children understand the items. Cronbach's alpha coefficient (α) was used to measure the internal consistency of the scales. Coefficient values above 0.70 were considered acceptable.^51^ The questionnaire included questions about the gender (I am a: girl/boy/non-gendered/other) and age of the children as well as a general anxiety scale for potential control variables. A question was included to assess if children had prior knowledge about climate change: Have you ever heard about climate change, including global warming, pollution, environmental degradation, extinction of certain species, glacier melting, ocean pollution or deforestation [yes/no]?

The present study was exploratory and examined whether the intervention had potential impacts on variables of interest. Thus, many outcomes were included in the analysis. The primary theoretical focus was on coping strategies (meaning-focused coping and avoidance-focused coping), as these were the primary targets of the intervention's theoretical framework. Indeed, the intervention was designed to enhance adaptive coping (meaning-focused coping) while reducing maladaptive coping (avoidance-focused coping). Problem-focused coping remained an exploratory variable. Eco-anxiety dimensions and self-determination were included since these variables could potentially be affected by adaptive coping strategies.

#### Coping with Climate Change

The primary outcome of coping with climate change was measured using the Coping with climate change scale, which has demonstrated good validity with a sample of children and youth from Sweden.^12,52^ The scale includes three subscales measured with a Likert-type scale from 1 (not at all true for me) to 5 (very true for me), where higher scores indicate higher usage of the coping strategy. The subscales included meaning-focused coping (6 items, α_pre_ = 0.685, α_post_ = 0.732, “I have faith in humanity; we can fix climate change”), avoidance coping (5 items, α_pre_ = 0.675, α_post_ = 0.660, “Nothing serious will happen during my lifetime because of climate change”), and problem-focused coping (3 items, α_pre_ = 0.723, α_post_ = 0.746, “I think about what I myself can do to help climate change”). This scale was translated into French (and back translated to English to verify that it represented well the original scale).

#### Eco-Anxiety

The eco-anxiety outcome was measured using the Hogg eco-anxiety scale, which has demonstrated good validity in adults.^8^ To our knowledge, no validated eco-anxiety scale for children existed when we launched the study. Thus, the scale was adapted to use simpler language, and each question was specified to relate directly to climate change. A definition of climate change based on the United Nations's definition^53^ was also added before the scale. A selection of 11 items measuring the frequency of feelings related to climate change on a 4-point Likert-type scale (0 = not at all, 3 = almost every day) was made, with higher scores suggesting higher levels of eco-anxiety. The scale included four subscales: affective eco-anxiety (4 items, α_pre_ = 0.823, α_post_ = 0.832, “worrying too much because of climate change”), rumination eco-anxiety (2 items, α_pre_ = 0.646, α_post_ = 0.672, “not being able to stop thinking of the future with climate change”), behavior eco-anxiety (3 items, α_pre_ = 0.644, α_post_ = 0.731, “having a hard time sleeping because of climate change”), and eco-anxiety about personal impact (2 items, α_pre_ = 0.669, α_post_ = 0.722, “feeling anxious about my responsibility to help the problem of climate change”). A total mean was also calculated to represent children's levels of global eco-anxiety (α_pre_ = 0.876, α_post_ = 0.890). This scale was translated to French using the method described above.

#### Basic Psychological Need Satisfaction

Basic psychological need satisfaction was assessed using the Self-determination at school scale,^54^ which measures the satisfaction of children's needs for competence, autonomy and affiliation within the school context. The scale comprises 9 items measured on a Likert-type scale from 0 (almost never) to 4 (almost always), with higher scores indicating higher levels of satisfaction. Questions included “I feel free to be myself” and “I feel like there is space for my ideas.” The internal consistency of the scale in the present sample was low at pre-test, and acceptable at post-test (α_pre_ = 0.687, α_post_ = 0.797).

#### Anxiety Symptoms

Since the link between eco-anxiety and general anxiety symptoms are unclear in children, a scale measuring the latter was added for exploratory purposes. Anxiety symptoms were measured using four selected items from the anxiety subscale of the Behavior assessment scale for children third edition for Canadian francophones (BASC-III; Reynolds & Kamphaus, 2015). Items were presented on a Likert-type scale from 0 (never) to 3 (almost always) to obtain a mean score, with higher scores indicating higher levels of anxiety. The psychometric qualities of this instrument are excellent and it is largely used in research and clinical settings with children.^55^ The internal consistency of this scale in the present sample was low (α_pre_ = 0.606, α_post_ = 0.699).

### Statistical Analysis

Univariate mixed-design analyses of variance (ANOVA) were conducted to compare mean differences between the two groups for each outcome using IBM SPSS v.29. Due to normality issues and outliers, the global scale of eco-anxiety, and sub-scale measures of affective eco-anxiety and behavior eco-anxiety were log-transformed (log10).^56^ After transformation, these variables met the assumption of normality and no outlier remained, except for behavior eco-anxiety, which was highly skewed towards the lowest score. This variable was not used in the analysis. The assumptions of homogeneity of variance (Levene's test) and homogeneity of covariances (Box's test) were met for all variables included in the analysis. Partial eta-square (η^2^) was used as a measure of effect size, where values around 0.01 were considered small effects, 0.06 as moderate effects, and 0.14 and above as large effects.^57^ Bonferroni adjustment was applied to correct for multiple comparisons. Post hoc analyses were also performed to control for general anxiety, gender, and children's age in the mixed ANOVAs.

## Results

### Participants

A total of four classes, including 102 children, were initially recruited. Seven children were absent from their class when the pre-test questionnaire was administered, and eight children were absent at the post-test measure time. Eighty-seven children who had filled out both the pre- and post-test questionnaires were included in the analysis, with 46 children in the experimental group and 41 in the control group. Given the clustered nature of the study, we conducted one-way ANOVAs to compare the class scores at pre-test. Two variables showed significant differences between classes (see Table 3). Post-hoc analyses suggested that classes 2 and 3 had significantly higher scores on both the total eco-anxiety and problem-focused coping scales. Given that class 2 was in the control group and class 3 in the experimental group, we considered that randomization balanced the groups and proceeded with an analysis that did not take into account clustering. Furthermore, the small number of clusters (k = 4) did not allow for a multilevel analysis that could take into account classroom as a random factor.^58^ Independent samples *t*-tests did not reveal any significant differences between groups at pre-test on any of the variables, indicating that groups were well balanced. Pre- and post-test means for each group can be found in Table 4. Knowledge about climate change was very high at baseline, with 100% of the sample indicating that they knew about climate change before the definition was provided in the questionnaire.

**Table 3.** One-Way ANOVA Comparing Class Scores at Pre-Test.

Variable	*F*	df	*p*
Meaning coping	0.991	3, 44.5	0.406
Problem coping	5.855	3, 44.5	**0**.**002**
Avoidance coping	0.360	3, 44.8	0.782
Overall eco-anxiety	2.834	3, 45.1	**0**.**049**
Rumination eco-anxiety	1.623	3, 44.1	0.198
Impact eco-anxiety	0.468	3, 45.2	0.706
Affective eco-anxiety	3.200	3, 45.4	0.032
Self determination	0.882	3, 44.4	0.458

**Table 4.** Mixed ANOVA Results.

Variable	M (SD)				F (η^2^)		
	Experimental		Control		Time	Group	Time X Group
	T1	T2	T1	T2			
Meaning-focused coping	3.63 (0.60)	3.63 (0.78)	3.68 (0.72)	3.68 (0.72)	0.000 (0.000)	0.09 (0.001)	0.006 (0.000)
Avoidance-focused coping	2.29 (0.86)	2.40 (0.81)	2.16 (0.78)	2.28 (0.94)	2.240 (0.026)	0.46 (0.006)	0.096 (0.001)
Problem-focused coping	3.05 (1.04)	2.97 (0.98)	2.79 (0.84)	2.63 (0.82)	2.795 (0.033)	3.241 (0.038)	0.342 (0.004)
Overall eco-anxiety	0.73 (0.64)	0.57 (0.52)	0.66 (0.41)	0.52 (0.43)	12.11*** (0.13)	0.07 (0.001)	0.02 (0.000)
Affective eco-anxiety	0.90 (0.87)	0.67 (0.65)	0.77 (0.57)	0.59 (0.85)	9.902** (0.104)	0.07 (0.001)	0.074 (0.001)
Rumination eco-anxiety	1.15 (0.86)	0.85 (0.70)	1.20 (0.79)	0.78 (0.69)	10.613** (0.112)	0.07 (0.001)	0.019 (0.000)
Impact eco-anxiety	0.73 (0.80)	0.67 (0.72)	0.65 (0.54)	0.70 (0.63)	0.001 (0.000)	0.06 (0.001)	0.509 (0.006)
Self-determination	2.48 (0.63)	1.60 (0.97)	2.70 (0.59)	1.81 (0.85)	0.165 (0.002)	4.113* (0.046)	0.270 (0.003)

*p < 0.05. **p < 0.01. ***p < 0.001.

T1: Pre-intervention, T2: Post-intervention

Effect size: Partial eta squared (η2)

### Outcomes and Estimation

Mixed ANOVA results revealed no significant interactions between the intervention and time on any of the variables, as shown in Table 4. A main effect of time was statistically significant between pre- and post-intervention for overall eco-anxiety (*F*(1, 85) = 12.106, *p* < .001, partial η^2^ = .125), affective eco-anxiety (*F*(1, 85) = 9.902, *p* < .002, partial η^2^ = .104), and rumination eco-anxiety (*F*(1, 84) = 10.613, *p* = 0.002, partial η^2^ = .112) with moderate effect sizes. Overall eco-anxiety, affective eco-anxiety and rumination eco-anxiety scores for the whole sample significantly decreased from pre- to post-test, regardless of group. Certain covariates linked to the measures outcomes were considered including age gender and anxiety symptoms at pre-test,^59^ but none of the covariates changed the pattern of results.

## Discussion

The present study aimed to evaluate the impact of a seven-week creative arts and philosophical inquiry intervention on the primary outcomes of children's coping with climate change and eco-anxiety dimensions, using a randomized cluster pilot trial. Results from mixed ANOVAs revealed no significant interaction effects of group and time, indicating that the hypotheses were not supported. This is surprising given that previous qualitative studies have found that artistic creation and philosophical inquiry could promote meaning-making, a more adaptive form of coping.^24,28^ While the intervention did not yield the expected benefits, it is important to point out that it did not heighten children's concerns by addressing climate change in the classroom. The intervention did not significantly reduce eco-anxiety, and it did not seem to increase the scores. This finding is promising, as parents and teachers may be concerned that discussing climate change and the related emotions could increase children's eco-anxiety.^42,44^ This pilot study lends support to the literature indicating that providing children with a safe and brave space to express their emotions about climate change does not cause additional distress when adapted approaches, such as artistic creation and philosophical inquiry, are employed.^39,60^ However, these null findings should be interpreted cautiously given the small sample size and design constraints. Furthermore, the eco-anxiety scores were very low in the present sample. This could suggest that children in this sample were not particularly concerned about the issue of climate change. This is not in line with previous qualitative results with children that find they are experiencing potential eco-anxiety.^24,28,50,61^ Scales that are better adapted to children could allow for a more valid measure of children's eco-anxiety. For example, the Basic Eco-anxiety Scale for Children and Adolescents (BEASCA) was validated after the end of the present study.^62^ Furthermore, measurements with greater sensitivity could be included in future studies, as there was limited room for improvement. For example, differentiating the negative consequences of eco-anxiety from habitual ecological worrying could facilitate an assessment of the various aspects and manifestations of eco-anxiety.^63^

Findings did not support the hypothesis that the intervention had an impact on coping with climate change. This could be partly due to the low measurement reliability of the scale. Meaning-focused coping may take a long time to develop, as it requires a change in the interpretation of the situation to render it more accessible. As such, meaning is created over a long period and may require more than seven weeks to develop, especially for younger children. Longitudinal data could give insight into this, as such changes could also be observed only over longer periods of time. Furthermore, meaning making is considered a complex construct to measure because it may be substantially different from one individual to the next.^64,65^ As such, meaning could be measured qualitatively using open-ended questions. Moreover, some elements could be integrated into the intervention to increase the creation of meaning. For example, the classes could conclude the workshops with an exhibition activity. This could allow them to share their experiences and artwork with their parents and stakeholders, which could generate meaning-making.^66^ While this was suggested to teachers, the end of the school year and other feasibility issues halted this initiative.

Results indicate a significant decrease in overall eco-anxiety, rumination eco-anxiety, and affective eco-anxiety scores at post-test across both groups (moderate effect sizes). Given that there was no significant interaction effect, these changes cannot be attributed to the intervention but rather apply to both groups. The decrease in overall eco-anxiety, rumination (cyclical thoughts about climate change), and affective eco-anxiety (feeling anxious and worried) could be due to natural changes over time. However, the results from the rumination eco-anxiety sub-scale should be interpreted with caution, as the internal consistency was low. Other factors outside the study, such as exposure to news or information about climate change, could also have affected both groups. For example, the pre-test questionnaire was completed by children in February, which coincided with record mild weather during the winter months, with temperatures reaching up to +5 degrees Celsius, causing rain and snow to melt completely from the ground, whereas normal nighttime temperatures should be around −25 degrees Celsius in Quebec, where the study was conducted.^67^ This unusual temperature could have led parents, teachers, and the media to discuss climate change and its impacts, making the entire sample of children more susceptible to experiencing eco-anxiety at pre-test, and these effects decreasing by post-test, with declined salience of the issue. The children's awareness of such consequences of climate change could be documented during assessment to gain a better understanding of their impacts on eco-anxiety. Other major phenomenon might have received more media and online coverage. While we did not systematically measure media exposure, the study period (February to April 2024) coincided with sustained international media coverage of humanitarian crises (eg, the war in Ukraine), domestic political concerns in Canada, and early spring weather transitions. These concurrent concerns may have gained children's attention during the study period, contributing to the observed pattern of natural habituation affecting both groups equally.

Furthermore, since both groups were in the same school, there could have been a diffusion effect or contamination across groups, whereby students in the control group could have been exposed to some of the intervention material.^68^ As such, this proximity between groups could have introduced bias, leading to an underestimation of the intervention's impact on the experimental group. It could also explain the reduction in overall eco-anxiety, affective and rumination eco-anxiety in both groups, as the control group may have been discussing climate change more with friends from other classrooms. This, coupled with the floor effects observed and sensitivity issues of the scale could have limited our ability to detect pre-to-post changes in the experimental group.

Problem-focused coping did not increase nor decrease in the experimental group after taking part in the intervention. It is possible that the school was already environmentally active, creating spaces for children to be engaged and act within the school. Measuring children's intention to act or empowerment levels could give a better understanding of their experiences, since children may have fewer opportunities to adopt pro-environmental behaviors.^69^ Furthermore, other forms of coping, such as emotion-focused coping, which includes searching for emotional support from others, were not included in the coping with climate change scale used in this study. This type of coping might be an intermediary form of coping before creating meaning, whereby children who feel validated and supported in their emotional experience could move towards creation of meaning instead of avoiding these emotions. Since emotional validation plays an important part in supporting people with eco-anxiety,^70,71^ active listening, empathy, normalizing of emotions, and support should be put forth in interventions with children.

### Self-Determination

While the intervention itself was rooted in self-determination theory and aimed to support the basic psychological needs of autonomy, competence and affiliation, the intervention did not have a significant impact on these variables. Bearing in mind that the psychometric properties of the measure in the present sample were not high, the present findings suggest that the intervention could include more elements that support the satisfaction of children's basic psychological needs. For example, promoting children's ability to self-direct their learning by choosing a subject related to climate change and completing their artistic creation on this subject could be integrated into the present intervention.^72^ The activities could include more elements of choice, and these could be made more explicit to children. For example, for the emotion wheel, children can be told to think of how they feel and choose emotions that relate to their own experiences. While this was the objective, it may not have been made clear enough, and children may not have felt autonomous in their choices. Further, additional challenging opportunities could be offered to the children to promote competence. For instance, including elaborate artistic methods, such as haikus and water painting, could be suggested. Research also suggests that autonomy is closely related to awareness, whereby people who are more mindful in the present moment may be better able to identify moments of choice and regulate their emotions.^73^ Integrating elements promoting mindfulness in children could allow them to be more aware of these moments. However, such findings have not been tested in children, and recent evidence suggests that mindfulness should be used with caution and under appropriate clinical supervision.^74,75^ Reviewing the activities to include more elements of choice to support autonomy, learning about different mediums, and practicing artistic abilities to support competence, as well as increasing the opportunities for feelings connected to others, could promote the satisfaction of these needs.^72^

### Limitations and Future Research

This pilot study provides important insight and lessons learned on conducting prevention research to support children's mental health in the context of climate change. The questionnaire package with children should be shorter, as some complained about the length and complexity of the questionnaire. To maintain children's attention, it is recommended that completion time remain under 30 min.^76,77^ This may have contributed to the low internal consistency for some of the scales. The low internal consistency could also partly be explained by the small number of items for some sub-scales. The findings from scales with low internal consistency (rumination eco-anxiety and self-determination) should be considered as preliminary evidence. One possibility to increase reliability would be to also ask teachers to fill out measures about their students. However, this would add significant amounts of work while they are already notably overworked and overwhelmed, and they might not be the best to observe internalizing difficulties like anxiety and eco-anxiety.^78^ While children are considered capable of providing good self-reports for their experiences,^79^ there are challenges to ensure that scales are adapted to children and can detect changes.^80^ Having research assistants fill out the questionnaire with one child at a time to ensure comprehension and validity could be an alternative, but this would require extensive resources and time with larger samples. As such, using mixed methods could offer a better understanding of children's experiences through both qualitative and quantitative measures. For example, open-ended questions about children's perception of climate change before and after the intervention could give insight into their emotions and coping in fewer constraining ways than questionnaires.

A limitation of this study is the sample size and analysis method that did not consider the clustered nature of the data. Given that some classrooms differed on problem-focused coping and overall eco-anxiety scores at pre-test highlights the relevance of including these considerations into the a-priori sample size calculation and analysis. With larger samples and at least 15 clusters, mixed models could be employed to consider the random factor of class.^58,81^ Nonetheless, the small sample size can be acceptable for a pilot study,^49^ and the randomization in the present study balanced the class effects. However, the statistical power accounting for the participant loss and missing data was reduced to 67% (post hoc calculation, 1-β = 0.67 with 87 participants). The preliminary effect size estimates from this pilot study provide valuable information for designing an adequately powered main trial. These estimates can be used to calculate sample size requirements, ensuring that future trials have sufficient power to detect the anticipated intervention effects while accounting for design complexities (classroom clustering, measurement reliability, potential contamination). Recruiting a larger population of children could also help gain a better understanding of the extent of eco-anxiety in this age-group given that scores were low in the present sample. Such larger and more diverse samples could allow for greater generalizability. The role of variables such as age, gender, general anxiety/depression, perceived direct impacts of climate change, and access to nature, could be explored. Furthermore, it is suggested that longitudinal data be included to provide information on the maintenance of change or the longer-term effects of the intervention. While it is a challenge to obtain longitudinal data when students change classes or graduate, this type of long-term information can provide much more insight into the impact of the intervention. Beginning the research project in the fall rather than in the winter could allow for follow-up measures at 6 months over the course of the same academic year.

The mechanisms underlying the intervention could also be explored in future studies. The present study did not specifically analyze potential pathways of change. Based on previous literature, one possible pathway might be that the intervention has an impact on maladaptive eco-anxiety through meaning-making and self-determination. However, the concept of eco-anxiety and coping in children remains understudied, and protective emotional factors are unclear. Nonetheless, some mediating variables could be included in future studies such as feeling empowered, feeling (emotionally) supported by adults, or emotional expression through the artwork. However, we suggest paying careful attention to the variables chosen given the limitation of questionnaire length with children.

Overall, the implementation process of this pilot study shows feasibility. The teachers and administration were all supportive and willing, helping with scheduling and sharing their excitement from start to finish. They said they would like to participate again and would appreciate being notified if there are other opportunities like this in the future. The engagement level in classes was high, and many students would greet the PhD student leading the activities with excitement, as was also documented in the qualitative acceptability study of this intervention.^50^ Teachers highly appreciated that an external person led the activity, as this provided them with respite. Furthermore, they also appreciated that someone with expertise on the topic of eco-anxiety would lead the activities, since they shared being uncomfortable with children's concerns. However, the sustainability of the intervention may be limited when an outside researcher implements workshops. Adequate preparation and training should be offered to teachers before integrating these activities, so they feel equipped to receive children's concerns. Future studies could explore the differences between receiving the intervention from an outside facilitator compared to trained teachers. Formal fidelity measures (eg, adherence checklists, quality rating scales) could also be incorporated in future trials to quantify implementation consistency and provide transparent documentation of protocol adherence.

The class-level clustering is convenient within school-based research, but a certain number of elements are not controllable, like students’ absences and classroom layout. For example, one classroom was composed of a single long table, on which students could not be far enough from each other to avoid seeing each other's questionnaire responses. This reduced privacy could have impacted on their responses. In future studies, the questionnaire could be completed over one week to reduce the loss of participants who are present only at the pre- or post-test administration, which is often for reasons outside the study (sickness, meetings, etc.) Another difficulty remained in recruiting enough classes to participate. Although more teachers were approached to attain the planned sample size, some of them were unable to fit the total seven hours of intervention into their curriculum schedules. Future research should aim to recruit a sufficient number of schools and classes to enhance power. Implementation strategies to minimize contamination could be considered such as recruiting from multiple schools to create geographic separation between control and experimental conditions, providing explicit guidance for teachers about limiting exposure to intervention content, and monitoring and documenting any contamination that occurs. For example, measuring perceived exposure to intervention content among control participants would provide empirical data on contamination levels. Evaluating the impact of interventions of various lengths (eg, 5 weeks vs 7 weeks) may also provide insight into the potential of brief interventions in this regard.

Schools should be contacted well in advance and provided with a timeline for the intervention's implementation before agreeing to participate. Control groups could also include different delivery formats (eg, teacher-led or researcher-led) and other interventions, such as bibliotherapy.^82^ The added value of each component of the intervention could also be evaluated by comparing art-only and philosophy-only groups to combined intervention groups. Nonetheless, studies on the topic of children's emotional experiences of climate change and coping have mostly used qualitative methods to obtain in-depth information about these experiences.^24,39,50^ The present pilot study provides information on transitioning to quantitative methods to gather complementary insight into the use of such approaches with children. The lessons learned and findings from the present study can guide future research to consider some of the important aspects discussed in this paper and apply some of the recommendations.

## Conclusion

The results from this pilot study do not support our initial hypothesis that participating in a creative arts and philosophical inquiry intervention would promote adaptive coping and reduce children's eco-anxiety in the experimental group when compared to the control group. While null findings from this pilot study should be interpreted cautiously, they provide preliminary evidence that there was no harm brought on by the intervention, and the natural reduction in global eco-anxiety, rumination, and affective eco-anxiety was not hindered by participating in the intervention. Although these non-significant results should be interpreted cautiously as an absence of evidence, eco-anxiety did not increase in the experimental group, suggesting that discussing climate change and expressing these emotions in a safe and creative context does not make children more anxious. Future longitudinal research is needed to better understand the impacts of this intervention with a larger sample and more sensitive measures. Before implementing a full-scale randomized controlled trial, it is recommended to conduct a larger feasibility study with more diverse samples, thereby increasing power and allowing to examine the potential moderating effects of age, gender and geographical location.

## Supplemental Material

sj-docx-1-css-10.1177_24705470261442334 - Supplemental material for Evaluating the Effectiveness of a Creative Arts and Philosophical Inquiry Intervention Rooted in Self-Determination Theory to Promote Adaptive Coping with Eco-Anxiety among Elementary School Children: A Pilot Randomized Cluster Trial Supplemental material, sj-docx-1-css-10.1177_24705470261442334 for Evaluating the Effectiveness of a Creative Arts and Philosophical Inquiry Intervention Rooted in Self-Determination Theory to Promote Adaptive Coping with Eco-Anxiety among Elementary School Children: A Pilot Randomized Cluster Trial by Terra Léger-Goodes, Catherine M. Herba, Jonathan Smith, David Lefrançois, Marc-André Éthier, Jasmine Piché and Catherine Malboeuf-Hurtubise in Chronic Stress
