# Supplementary material for: Evaluating the Effectiveness of a Creative Arts and Philosophical Inquiry Intervention Rooted in Self-Determination Theory to Promote Adaptive Coping with Eco-Anxiety among Elementary School Children: A Pilot Randomized Cluster Trial
Source: Chronic Stress (Thousand Oaks). 2026 Apr 17;10:24705470261442334. doi: 10.1177/24705470261442334 (PMC13092825; doi:10.1177/24705470261442334)
Supplement: sj-docx-1-css-10.1177_24705470261442334 - Supplemental material for Evaluating the Effectiveness of a Creative Arts and Philosophical Inquiry Intervention Rooted in Self-Determination Theory to Promote Adaptive Coping with Eco-Anxiety among Elementary School Children: A Pilot Randomized Cluster Tria [file sj-docx-1-css-10.1177_24705470261442334.docx]

**Supplementary Table 1.** Independent samples *t*-test comparing the experimental group and the control group scores at pre-test

| Variable | *t* | df | *p* | Mean difference (control - exp.) |
| --- | --- | --- | --- | --- |
| Meaning coping | 0.315 | 85 | 0.754 | 0.0445 |
| Problem coping | 1.257 | 84 | 0.212 | -0.2591 |
| Avoidance coping | 0.732 | 84 | 0.466 | -0.1310 |
| Total eco-anxiety | 0.174 | 85 | 0.863 | -0.0110 |
| Rumination eco-anxiety | 0.267 | 84 | 0.790 | 0.0478 |
| Impact eco-anxiety | 0.553 | 85 | 0.582 | -0.0819 |
| Affective eco-anxiety | 0.353 | 85 | 0.725 | -0.0296 |
| Self determination | 1.66 | 85 | 0.100 | 0.2188 |

| **Supplementary Table 2.** One-Way ANOVA comparing the class scores at pre-test   \| Variable \| *F* \| df \| *p* \| \| --- \| --- \| --- \| --- \| \| Meaning coping \| 0.991 \| 3, 44.5 \| 0.406 \| \| Problem coping \| 5.855 \| 3, 44.5 \| **0.002** \| \| Avoidance coping \| 0.360 \| 3, 44.8 \| 0.782 \| \| Total eco-anxiety \| 2.834 \| 3, 45.1 \| **0.049** \| \| Rumination eco-anxiety \| 1.623 \| 3, 44.1 \| 0.198 \| \| Impact eco-anxiety \| 0.468 \| 3, 45.2 \| 0.706 \| \| Affective eco-anxiety \| 3.200 \| 3, 45.4 \| 0.032 \| \| Self determination \| 0.882 \| 3, 44.4 \| 0.458 \| |
| --- | --- | --- | --- | --- | --- | --- | --- | --- | --- | --- | --- | --- | --- | --- | --- | --- | --- | --- | --- | --- | --- | --- | --- | --- | --- | --- | --- | --- | --- | --- | --- | --- | --- | --- | --- | --- |

| **Supplementary Table 3.** Tukey Post-Hoc Test – problem coping at pre-test | | | | | |
| --- | --- | --- | --- | --- | --- |
|  |  | 4 | 2 | 1 | 3 |
| 4 | Mean difference | — | -0.509 | 0.121 | -0.958 |
|  | p-value | — | 0.225 | 0.965 | 0.002 |
| 2 | Mean difference |  | — | 0.631 | -0.449 |
|  | p-value |  | — | 0.108 | 0.367 |
| 1 | Mean difference |  |  | — | -1.079 |
|  | p-value |  |  | — | <.001 |
| 3 | Mean difference |  |  |  | — |
|  | p-value |  |  |  | — |

 Note: Classes 1 and 2 = control group; cases 3 and 4 = experimental group

**Supplementary Figure 1.** Plot of means at pre-test for the problem-coping variable with a 95% confidence interval.

| **Supplementary Table 4.** Tukey Post-Hoc Test – total eco-anxiety at pre-test | | | | | |
| --- | --- | --- | --- | --- | --- |
|  |  | **4** | **2** | **1** | **3** |
| **4** | **Mean difference** | — | -0.150 | -0.0709 | -0.259 |
|  | **p-value** | — | 0.307 | 0.825 | 0.013 |
| **2** | **Mean difference** |  | — | 0.0788 | -0.110 |
|  | **p-value** |  | — | 0.808 | 0.609 |
| **1** | **Mean difference** |  |  | — | -0.188 |
|  | **p-value** |  |  | — | 0.133 |
| **3** | **Mean difference** |  |  |  | — |
|  | **p-value** |  |  |  | — |

 Note: Classes 1 and 2 = control group; cases 3 and 4 = experimental group

**Supplementary Figure 2.** Plot of means at pre-test for the total eco-anxiety variable with a 95% confidence interval.
